# Surface Erosion–Corona Synergistic Degradation Characteristics and Material Adaptability of High-Temperature Vulcanized Silicone Rubber in Desert Regions

**DOI:** 10.3390/polym18172051

**Published:** 2026-08-24

**Authors:** Jianghai Geng, Jingwei Li, Lingling Li, Ping Wang, Zhengbo Tian, Wei Liu

**Affiliations:** 1 Hebei Provincial Key Laboratory of Power Transmission Equipment Security Defense, North China Electric Power University, Baoding 071003, China; gengjh@ncepu.edu.cn (J.G.); 220242213042@ncepu.edu.cn (J.L.); 220242213078@ncepu.edu.cn (L.L.); 2 State Grid Electric Power Research Institute, Wuhan 430000, China; 3 State Grid Xinjiang Electric Power Research Institute, Urumqi 830000, China

**Keywords:** HTV silicone rubber, windblown sand erosion, corona aging, synergistic effect

## Abstract

The harsh sandstorm and the strong electric fields generated by operating transmission lines in desert regions can readily lead to performance degradation and shortened service life of high-temperature vulcanized (HTV) silicone rubber (SR) composite insulators. To screen HTV silicone rubber materials suitable for desert applications, this study conducted aging tests under windblown sand erosion, corona aging, and their combined synergistic effect on three HTV silicone rubber materials. By comparing the evolution of surface morphology and chemical structure under single-factor and combined aging conditions, the acceleration mechanism of erosion on corona aging was analyzed. Further comparison of the performance degradation of the three materials after identical aging durations revealed that the higher the hardness of HTV silicone rubber, the stronger its resistance to erosion, and it retains superior corona aging resistance after erosion. Under combined erosion and corona aging, the high-hardness S3 HTV silicone rubber maintained an intact surface micro-morphology and molecular chain structure, with a smaller reduction in elongation at break, sustained high volume and surface resistivity, and no noticeable deterioration in dielectric properties. A comprehensive evaluation demonstrates that the high-hardness S3 silicone rubber exhibits superior environmental adaptability in desert regions.

## 1. Introduction

Currently, the abundant wind and solar energy resources in desert regions are being increasingly exploited and utilized [1,2,3]. The vast geographical expanse of these areas compels local power grids to rely heavily on composite insulators made of high-temperature-vulcanized (HTV) silicone rubber (SR), rendering these devices vital for safeguarding the secure and reliable operation of power transmission lines [4,5]. However, the unique and harsh environmental conditions in desert regions pose severe challenges to the long-term stable operation of these insulators [6,7,8]. Under strong electric fields, composite insulators are continuously subjected to the impact of windblown sand, which causes the gradual aging of the HTV silicone rubber material, shortens its effective service life, and poses a severe risk to the secure operation of power transmission corridors in desert regions.

The evolution of silicone rubber material properties following corona aging under certain complex environmental conditions has been systematically investigated. Lu et al. [9] investigated the corona aging of high-temperature vulcanized silicone rubber under high humidity and thermal cycling, finding that these conditions caused severe surface deterioration, a decline in mechanical properties, and a loss of insulating capability. Zeng et al. [10] studied the coupling effect of ultraviolet B radiation and corona aging in plateau environments, and their results indicated that intense ultraviolet B exposure accelerated the propagation of surface cracks in HTV silicone rubber. Bi et al. [11] conducted 100 h corona aging tests under different humidity, salt spray, and haze conditions, and found that with increasing relative humidity, the degree of surface oxidation deepened and the insulation performance declined.

Corona aging primarily causes chemical corrosion of silicone rubber by altering its molecular structure, whereas windblown sand flows mainly induce physical erosion by compromising the structural integrity of the material [12,13,14]. Liu et al. [15] found that scratches, pits, and flaky spalling appeared on the surface of silicone rubber after abrasive wear. Besztercey et al. [16] experimentally determined the erosion rates of three silicone rubber materials with different mechanical properties under various particle velocities and analyzed the erosion damage mechanisms of the different silicone rubber materials. The above findings have predominantly examined the mechanisms of erosion-induced damage to materials, with inadequate focus on how silicone rubber’s performance degrades under these conditions. Consequently, these conclusions are of limited value in effectively guiding material selection in engineering practice.

In desert regions, severe weather involving strong windblown sand increases the surface roughness of composite insulators on transmission lines and causes the sheds to become worn and thinner [17]. Simultaneously, corona aging further accelerates the decline in both electrical and mechanical performance of composite external insulation materials, posing a serious risk to the security and stability of power transmission corridors [18,19,20]. Existing studies have explored the synergistic aging effects of corona discharge coupled with multiple environmental factors such as ultraviolet radiation, temperature and humidity variations, and atmospheric pollution, confirming the complex coupling interactions among various stresses. However, none of these works have addressed wind–sand erosion, a typical stress in desert regions. The aging mechanism of silicone rubber materials under the combined action of wind–sand erosion and corona discharge remains unclear, making it difficult to provide a basis for the scientific selection of composite insulators for desert areas. Therefore, it is imperative to introduce wind–sand erosion into the corona aging system, establish combined sand erosion–corona aging conditions, elucidate the degradation mechanism of silicone rubber materials under the synergistic action of corona aging and wind–sand erosion, and screen silicone rubber materials suitable for desert regions that possess both erosion resistance and aging resistance.

To address the above issues, this study conducted aging tests under single windblown sand erosion, single corona aging, and their synergistic effect on three HTV silicone rubber materials with different formulations, aiming to investigate the accelerating effect of sand erosion damage on corona aging. By testing the physicochemical properties, multi-scale mechanical properties, and electrical properties of the aged materials, the degree of performance degradation of the different materials was compared and analyzed, and their resistance to erosion wear and their ability to withstand corona aging after damage were evaluated, so as to identify the HTV silicone rubber material suitable for desert regions. The research findings can establish a theoretical foundation for selecting materials for composite insulators in desert regions, helping to extend the operational life of insulators and ensure the stable operation of renewable energy transmission corridors.

## 2. Materials

### 2.1. HTV Silicone Rubber Formulation

The three high-temperature vulcanized silicone rubber materials selected in this study are all commercial formulations commonly used in the manufacturing of composite insulators. They were provided by State Grid Xiangyang Insulator Co., Ltd. (Xiangyang, China) and are designated as S1, S2, and S3, respectively. The formulations of the materials are shown in Table 1, and the initial performance parameters are presented in Table 2. The S1 material has a Shore A hardness of 72.1, which is relatively low, and its initial mechanical properties are superior. The S2 and S3 materials have Shore A hardness values of 79.2 and 84.6, respectively, which are comparatively higher, and their initial mechanical properties are lower than those of the S1 material. The samples were flat plate specimens with dimensions of 160 mm × 100 mm × 1.8 mm, and their formulations and preparation methods were identical to those of the finished insulator sheath.

Before the tests, the surfaces of the HTV silicone rubber specimens were cleaned with anhydrous ethanol (Tianjin Shengxinyuan Chemical Co., Ltd. Tianjin, China) and air-dried at room temperature prior to conducting the sand erosion or corona aging tests. For specimens subjected to the combined aging test, the surfaces were wiped again with anhydrous ethanol after the erosion test to remove attached sand particles, thereby preventing them from interfering with the physicochemical properties of the HTV silicone rubber material during the subsequent corona aging test.

### 2.2. Abrasive Particles

**Figure 1:**
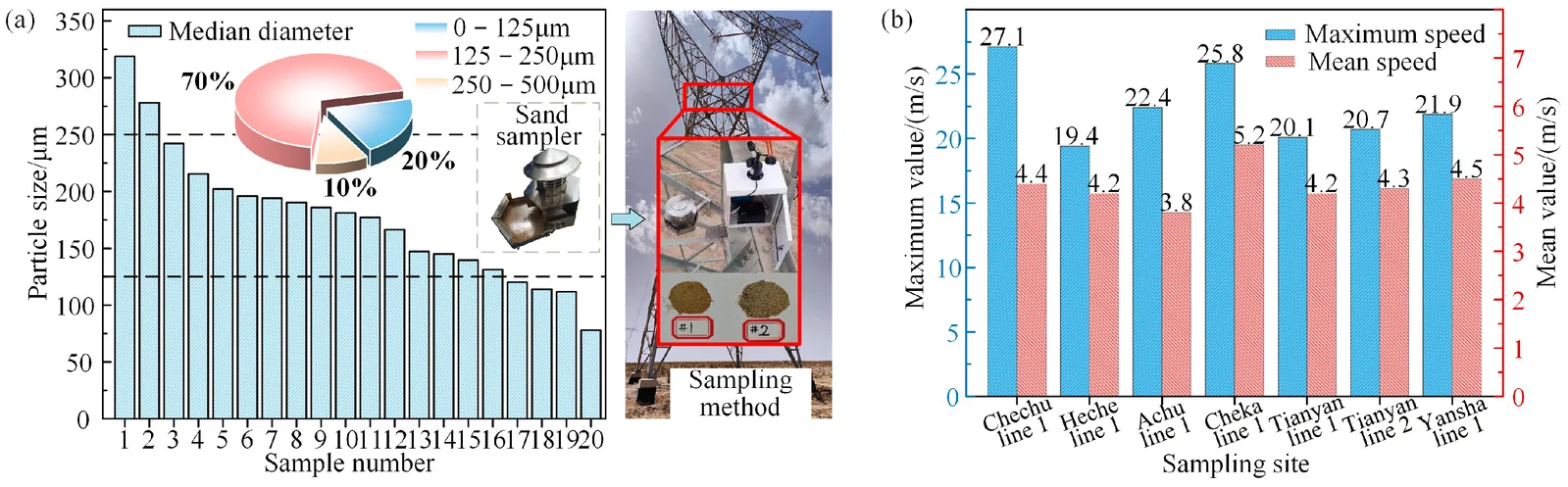
Survey results of wind–sand parameters: (**a**) sampling method and median particle size range; (**b**) the maximum and average wind speed.

Twenty transmission towers along transmission lines near the Taklamakan Desert and the Qaidam Desert were selected as field sampling sites [21]. Windblown sand particle collection devices and wind speed recording instruments were installed on the horizontal angle steel of the towers at the same height as the line insulators. The sampling period spanned from March to May, when wind–sand activities are most frequent [22]. Particle size analysis was conducted on the collected sand samples, and the median particle size at each sampling site is shown in Figure 1a. The median particle size at approximately 70% of the sampling sites fell within the range of 125~250 μm. Accordingly, sand with a particle size range of 125~250 μm was selected for use in the sand erosion simulation tests. Figure 1b presents the maximum and average wind speeds during the sampling period. Among all the sampling sites, a maximum wind speed of 27.1 m/s was recorded at the Chechu line I due to its unique geographical environment. To analyze the material performance degradation under strong wind–sand conditions, the simulated wind–sand erosion test was conducted at a constant wind speed of 30 m/s for a duration of 60 min.

## 3. Methods

### 3.1. Windblown Sand Erosion Test Apparatus and Method

**Figure 2:**
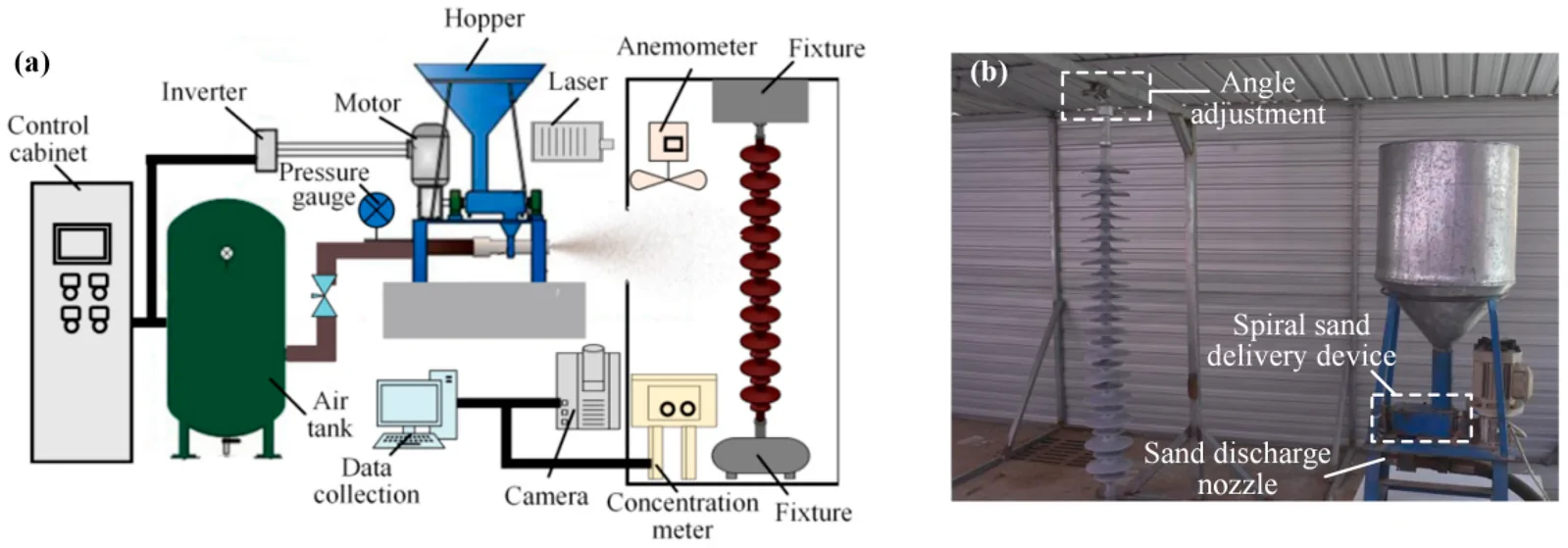
Simulated sand erosion test device: (**a**) schematic diagram, (**b**) physical photograph.

The air-jet sand-blasting-type simulated wind–sand erosion test apparatus used in this study is shown in Figure 2. During the test, the air supply system, consisting of a screw air compressor and an air storage tank, continuously delivers a stable airflow, while the airflow velocity at the nozzle is regulated by a pressure regulating valve. When the sand discharge valve is opened, the sand particles in the hopper fall into the screw feeder below and are then conveyed into the sand supply pipe. The high-speed airflow is ejected from the nozzle, forming a wind–sand two-phase flow that meets the test requirements within the test chamber. The placement angle of the HTV silicone rubber specimen is adjusted via a fixture, with the erosion angle set at 45°.

### 3.2. Corona Aging Test Apparatus and Method

**Figure 3:**
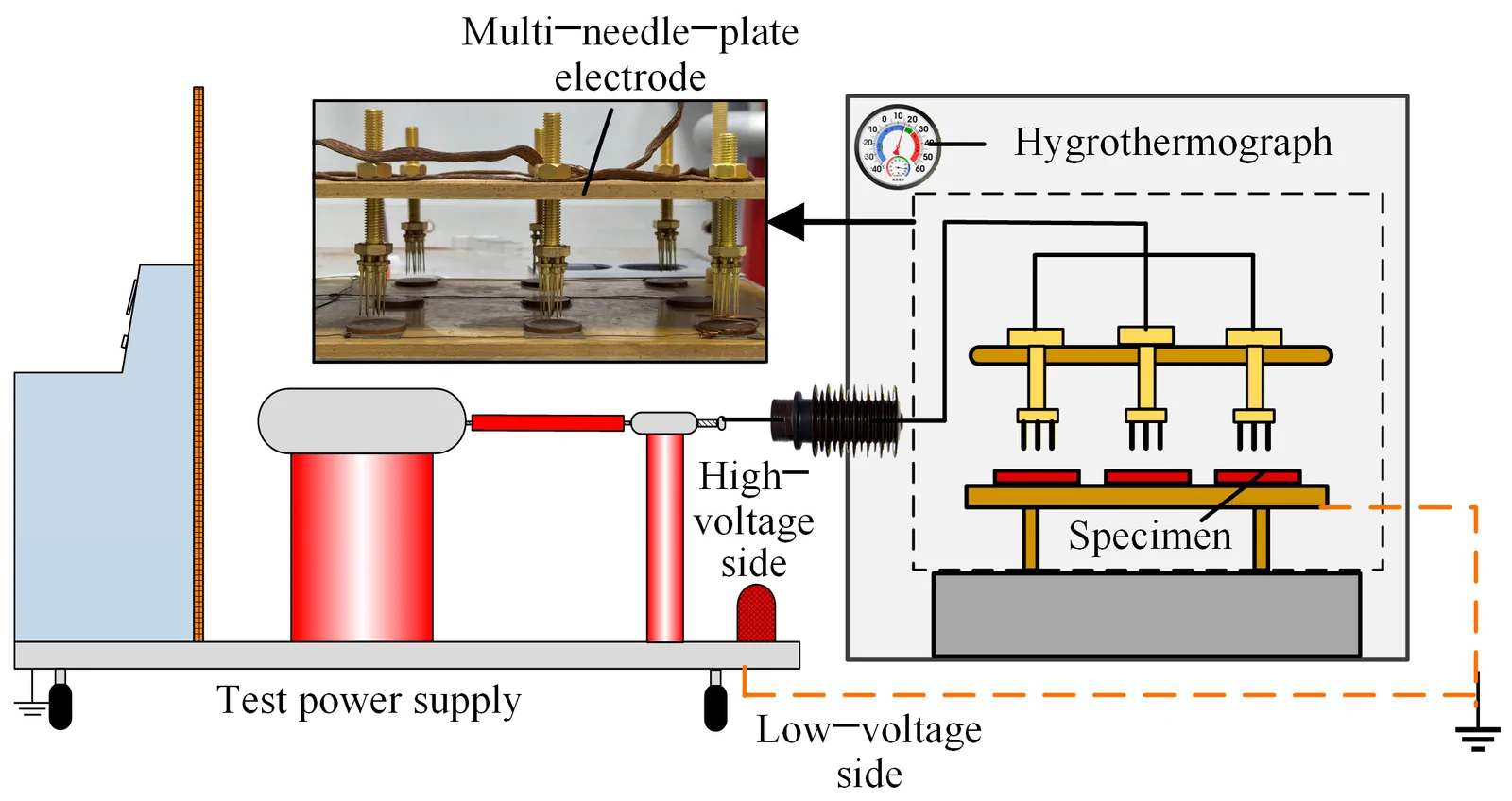
AC corona aging test platform.

The AC corona aging test platform is shown in Figure 3. A multi-needle-to-plate configuration is adopted for discharge. The corona electrode consists of seven needles, with six needles evenly distributed in a regular hexagon around a central needle. The tip curvature radius is approximately 0.2 mm. During the test, the applied voltage is 8 kV, the gap between the needle tips and the HTV silicone rubber specimen is set to 2 mm, and the duration is 500 h.

### 3.3. Experimental Procedures

The HTV silicone rubber specimens were divided into three groups: one group underwent the combined erosion–corona aging test, while the other two groups underwent single windblown sand erosion and single corona aging tests, respectively. The combined aging test was conducted in a stepwise manner. After pretreatment, the specimens were first subjected to windblown sand erosion and then to corona aging. Subsequently, the physicochemical properties, mechanical properties, and electrical properties of each group of specimens were tested and comparatively analyzed. The specific test methods are described as follows.

A Pi85 nanomechanical test system (Bruker, Berlin, Germany) was employed to conduct nanoindentation, allowing the assessment of nano-hardness and elastic modulus, as well as the characterization of the micro-mechanical properties of the HTV silicone rubber materials. The maximum indentation load applied during the test was 1 mN, and the holding time was 10 s.

A VERTEX 70 Fourier transform infrared spectrometer (Bruker, Ettlingen, Germany) was used to characterize the surface chemical structure of the silicone rubber specimens before and after aging, in the attenuated total reflection (ATR-FTIR) mode. The ATR crystal used was single crystal silicon, with the number of scans set to 32, a spectral resolution of 4 cm^−1^, and a wavenumber range of 4000–600 cm^−1^.

The tensile strength and elongation at break of the HTV silicone rubber specimens were measured using a universal testing machine (Dongguan Huanyi Instrument Technology Co., Ltd., Dongguan, China, model HYH-1201, speed range 0.1–500 mm/min), in accordance with the method specified in ISO 37:2005 [23] (Rubber, vulcanized or thermoplastic—Determination of tensile stress–strain properties). Each test was repeated five times, and the final results were expressed as the median.

Hydrophobicity tests were conducted on the HTV silicone rubber specimens in accordance with the method described in IEC/TS 62073:2016 [24] for evaluating the hydrophobicity of insulator surfaces. During the test, five positions were selected in the areas with more severe aging for measurement, and the final result was expressed as the average value of the measured data at each point.

In accordance with IEC/TS 62073:2016, the static contact angle of the silicone rubber specimens was measured using a contact angle tester (Shandong Jiuzhang Scientific Instrument Co., Ltd., Weifang, China, measuring range 0–180°) to evaluate their hydrophobicity. During the test, five positions in the more severely aged areas were selected for measurement, and the final result is expressed as the average of the data from each measurement point.

After corona aging treatment, the specimens were allowed a sufficient recovery period. In accordance with IEC 62631-3-1:2016 [25], the surface resistivity was measured using a surface resistivity tester (Beijing Guance Jingdian Instrument Equipment Co., Ltd., Beijing, China, model GEST-121). Three replicate specimens were prepared for each test, and the final results are expressed as the median. In accordance with ASTM D150-22 [26], the dielectric constant and dielectric loss of the silicone rubber specimens were measured using a dielectric constant and dielectric loss tester (Beijing Aerospace Weichuang Equipment Technology Co., Ltd., Beijing, China). All tests and measurements in this study were conducted in an environment at a temperature of 25 °C and a relative humidity of 40%.

## 4. Results and Discussion

### 4.1. Performance Changes Under Sand Erosion

#### 4.1.1. Micro-Morphology

**Figure 4:**
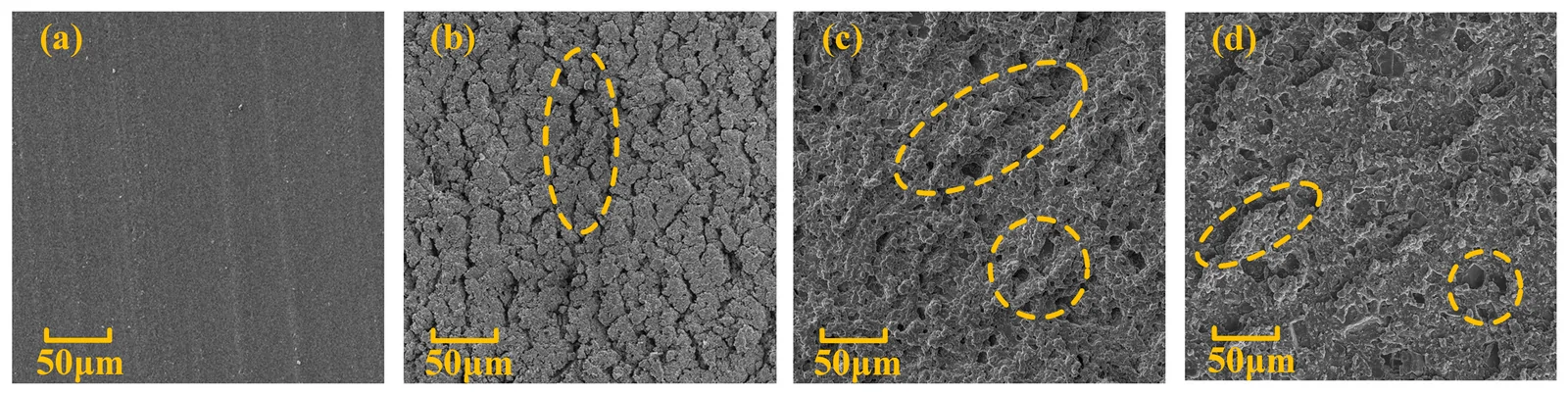
The microstructure of different silicone rubber before and after erosion: (**a**) before erosion; (**b**) S1; (**c**) S2; (**d**) S3.

The micro-morphology of the three HTV silicone rubber specimens before and after erosion is shown in Figure 4. The untreated specimens exhibit a smooth surface and intact structure. After sand erosion, distinct differences in micro-morphology emerge due to the varying wear mechanisms of HTV silicone rubber with different hardness levels. For the harder S3 silicone rubber, the impact of sand particles primarily manifests as a cutting action, leading to the formation of pits on the material surface. Under continuous erosion, oblique cracks initiate near the pits, and subsequent crack propagation eventually causes flaking of the material. In contrast, the softer S1 silicone rubber is prone to developing numerous deep and elongated cracks during erosion, with the crack network evolving rapidly and resulting in a fish-scale-like tearing of the material surface. The S2 material, with hardness between that of S3 and S1, exhibits slightly fewer and shorter cracks than S1, and a degree of material flaking slightly greater than that of S3. It is evident from the comparison that the S3 material has fewer surface cracks and relatively mild erosion damage, whereas the low-hardness materials display dense and deep cracks with significantly more severe damage.

#### 4.1.2. Micro-Mechanical Properties

**Figure 5:**
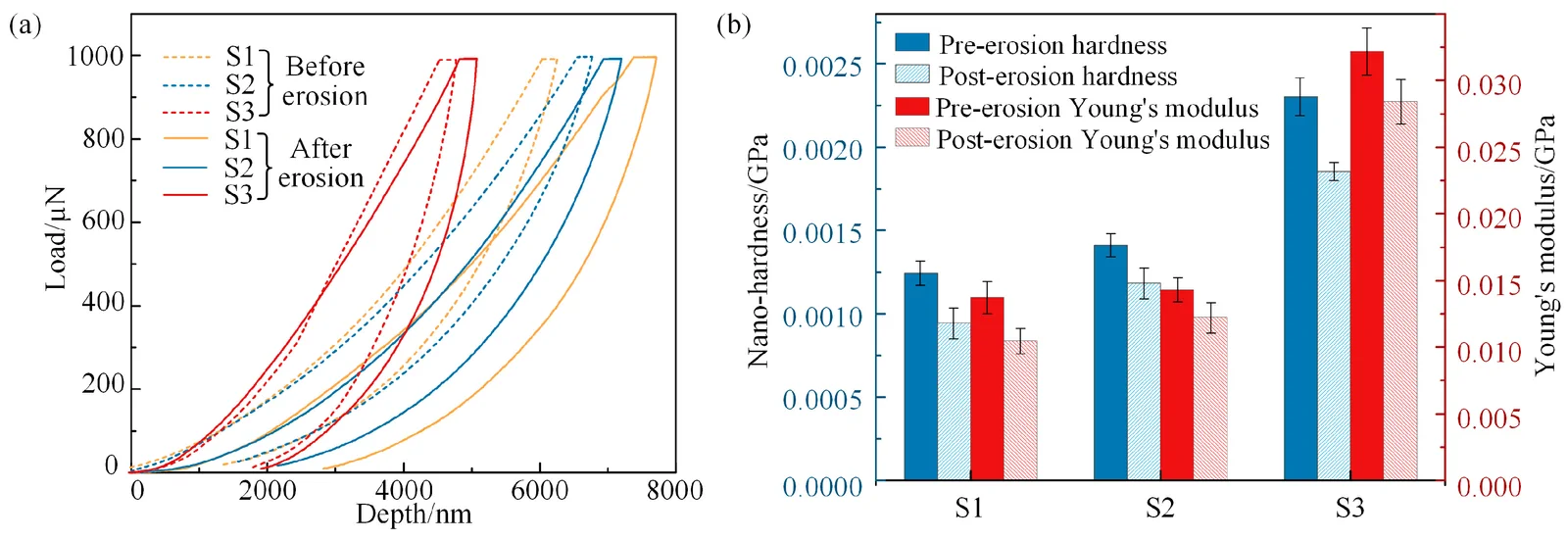
Micro-mechanical properties of different silicone rubber materials before and after wind–sand erosion: (**a**) load–displacement curves; (**b**) nano-hardness and young’s modulus.

Nano-indentation is a micro-scale method for characterizing the mechanical properties of materials. During the test, a diamond indenter is pressed into the material surface under a constant load, and the load–displacement curve during the loading and unloading process is recorded, as shown in Figure 5a. The nano-hardness and elastic modulus of the material were calculated based on the Oliver–Pharr method [27], and the results are presented in Figure 5b, thereby evaluating the changes in the surface mechanical properties of the HTV silicone rubber induced by windblown sand erosion.

After windblown sand erosion, the nano-hardness and Young’s modulus of all three materials decreased, indicating degradation of their surface micro-mechanical properties. Specifically, the harder S3 material retained 80.62% and 88.39% of its initial nano-hardness and Young’s modulus, respectively, while the softer S1 material, which suffered the most severe wear, retained only 75.90% and 76.29% of these initial values. Combined with the micro-morphological changes in the HTV silicone rubber surfaces before and after erosion, it can be inferred that under the local stress induced by the impact of windblown sand flows, the surface structure of the HTV silicone rubber becomes loose, the interfacial bonding between the filler and the HTV silicone rubber matrix fails, and micro-cracks develop around the filler particles. When the indenter is pressed in, these micro-defects coalesce and propagate, making the HTV silicone rubber more easily compressed, which ultimately manifests as a decline in mechanical properties.

#### 4.1.3. Macro-Mechanical Properties

**Figure 6:**
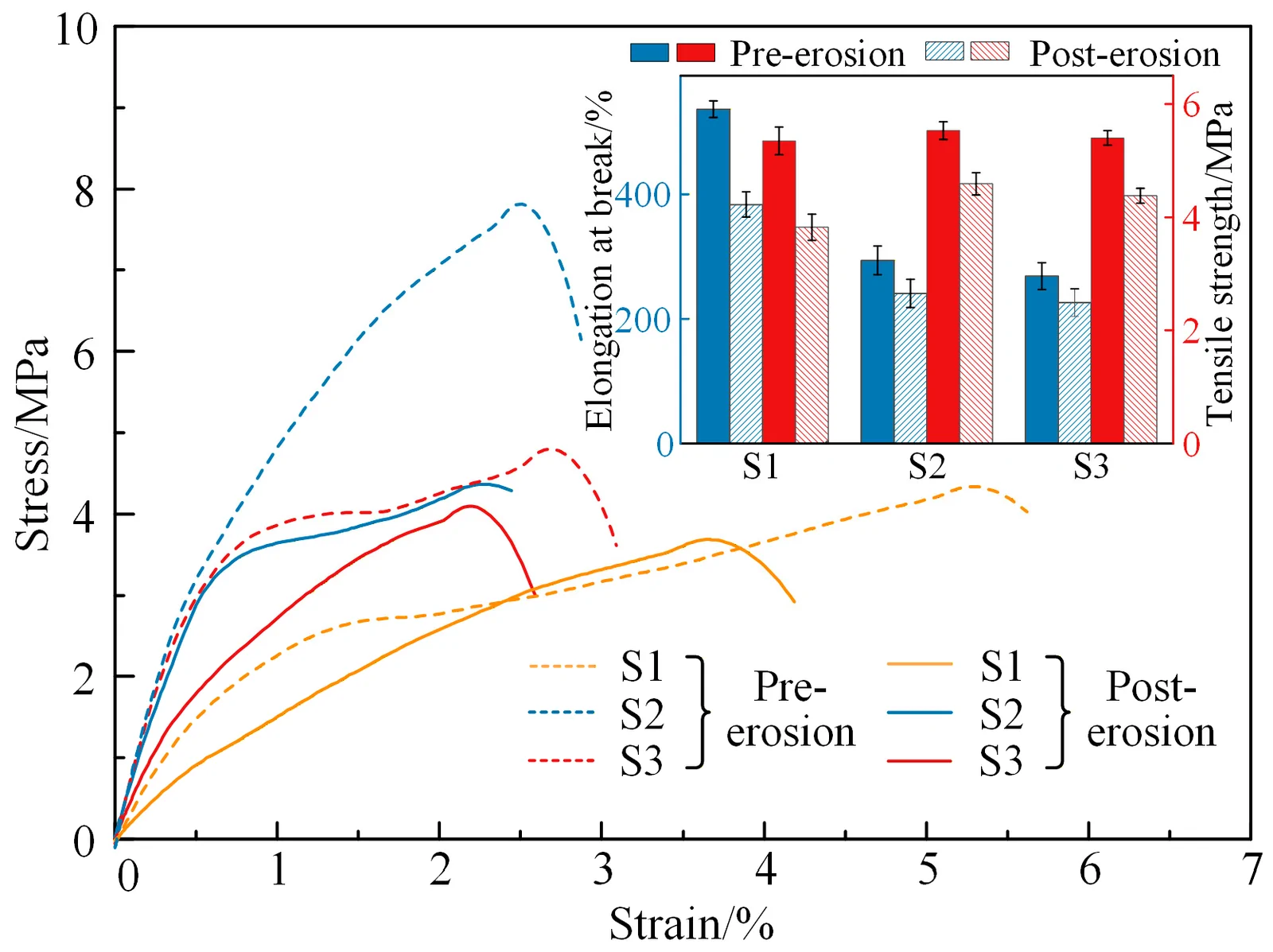
The stress–strain curve of silicone rubber material before and after erosion test.

To evaluate the effect of windblown sand erosion on the macro-mechanical properties of HTV silicone rubber materials, quasi-static tensile tests were conducted on the specimens before and after erosion. The specimen was a standard dumbbell-shaped, with a total length of 115 mm, an end width of 25 mm, a narrow section width of 6 mm, and a thickness of 3 mm. Figure 6 shows the stress–strain curves as well as the tensile strength and elongation at break of the different HTV silicone rubber materials before and after wind–sand erosion. After wind–sand erosion, the elongation at break of the lower-hardness S1 material decreased from 536.24% before erosion to 383.54%, and the tensile strength decreased from 5.35 MPa to 3.82 MPa, representing reductions of 28.48% and 28.60%, respectively. In contrast, the higher-hardness S3 material exhibited the smallest decline in mechanical properties: its elongation at break decreased from 308.0% to 240.91%, and its tensile strength decreased from 5.40 MPa to 4.38 MPa, representing reductions of 21.78% and 18.89%, respectively.

Based on the micro-morphology of the material surfaces after erosion, the surface scratches and micro-cracks generated by sand particle erosion act as stress concentration sites during the tensile process, readily initiating and propagating cracks and thereby accelerating material fracture. The high-hardness HTV silicone rubber material S3 exhibits a cutting wear mechanism under the impact of high-speed sand particles, with material damage mainly characterized by flaky peeling, and its structural integrity is largely preserved. In contrast, although the HTV silicone rubber material S1 with a lower HTV silicone rubber content possesses good initial mechanical properties, it develops dense cracks and fissures under sand particle erosion, resulting in significant reductions in elongation at break and tensile strength.

### 4.2. Performance Changes Under the Synergistic Effect of Erosion and Corona

#### 4.2.1. Surface Morphology and Elemental Distribution

**Figure 7:**
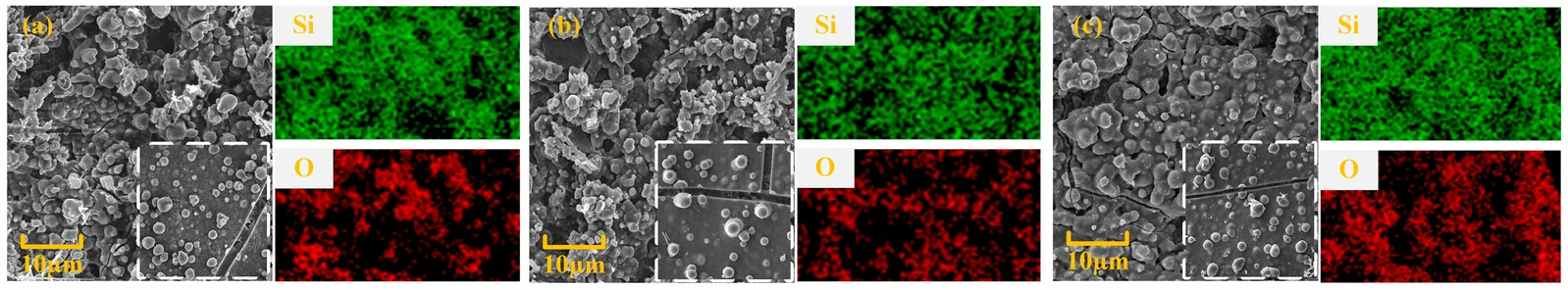
SEM and EDS images after single corona aging and erosion–corona aging: (**a**) S1; (**b**) S2; (**c**) S3.

The SEM images and EDS results of the three silicone rubber materials after undergoing windblown sand erosion first and then corona aging are shown in Figure 7. After aging treatment, voids, cracks, and flocculent precipitates appeared on the surfaces of all materials. The surface of the S3 material was relatively intact, with only a small number of cracks and flaking, whereas the S1 material was evidently loose and porous, exhibiting denser cracks and voids along with numerous flocculent precipitates on the surface. To analyze the effect of windblown sand erosion on corona aging, the micro-morphology of the same HTV silicone rubber specimens subjected only to corona aging was compared. The results show that under corona aging alone, the surface primarily exhibited localized cracking and sparse spherical stacked structures; in contrast, after the combined action of erosion and corona, the surface voids, cracks, and flocculent precipitates increased significantly, and the overall damage morphology became more severe.

Further analysis of the elemental distribution on the surfaces of the HTV silicone rubber specimens at each stage is presented in Table 3. Compared with the untreated specimens, after the combined aging test, the Si content on the surfaces of the S1, S2, and S3 specimens increased by 78.41%, 70.45%, and 72.31%, respectively; the O content increased by 56.31%, 67.31%, and 52.40%; whereas the C content decreased by 70.08%, 75.40%, and 65.78%. This trend is consistent with the finding of Bao et al. [28] that corona aging leads to a decrease in C content and a relative enrichment of O and Si on the silicone rubber surface. However, owing to the preceding wind–sand erosion, the changes in elemental content after combined aging are more pronounced. A substantial amount of organic components was lost from the material surface, and the resulting spherical stacked structures were mainly composed of Si and O elements.

In the initial state of HTV silicone rubber, the Si atoms are located in the middle region of the cross-linked molecular chains. Under the effect of corona aging, the polymer backbone undergoes scission, exposing some Si elements on the material surface and leading to an increase in Si content in the EDS spectra. After the backbone is broken, the organosilicon chain segments simultaneously undergo oxidation reactions, generating a large amount of inorganic silicon oxides, and the proportion of O element rises substantially. As corona aging progresses further, the Si-CH_3_ side chains are cleaved, and the C element in the methyl groups escapes into the air in the form of CO or CO_2_, resulting in a decrease in C content on the material surface. The S1 specimen exhibited a greater reduction in C content, indicating severe damage to its chemical structure.

#### 4.2.2. Physicochemical Properties

**Figure 8:**
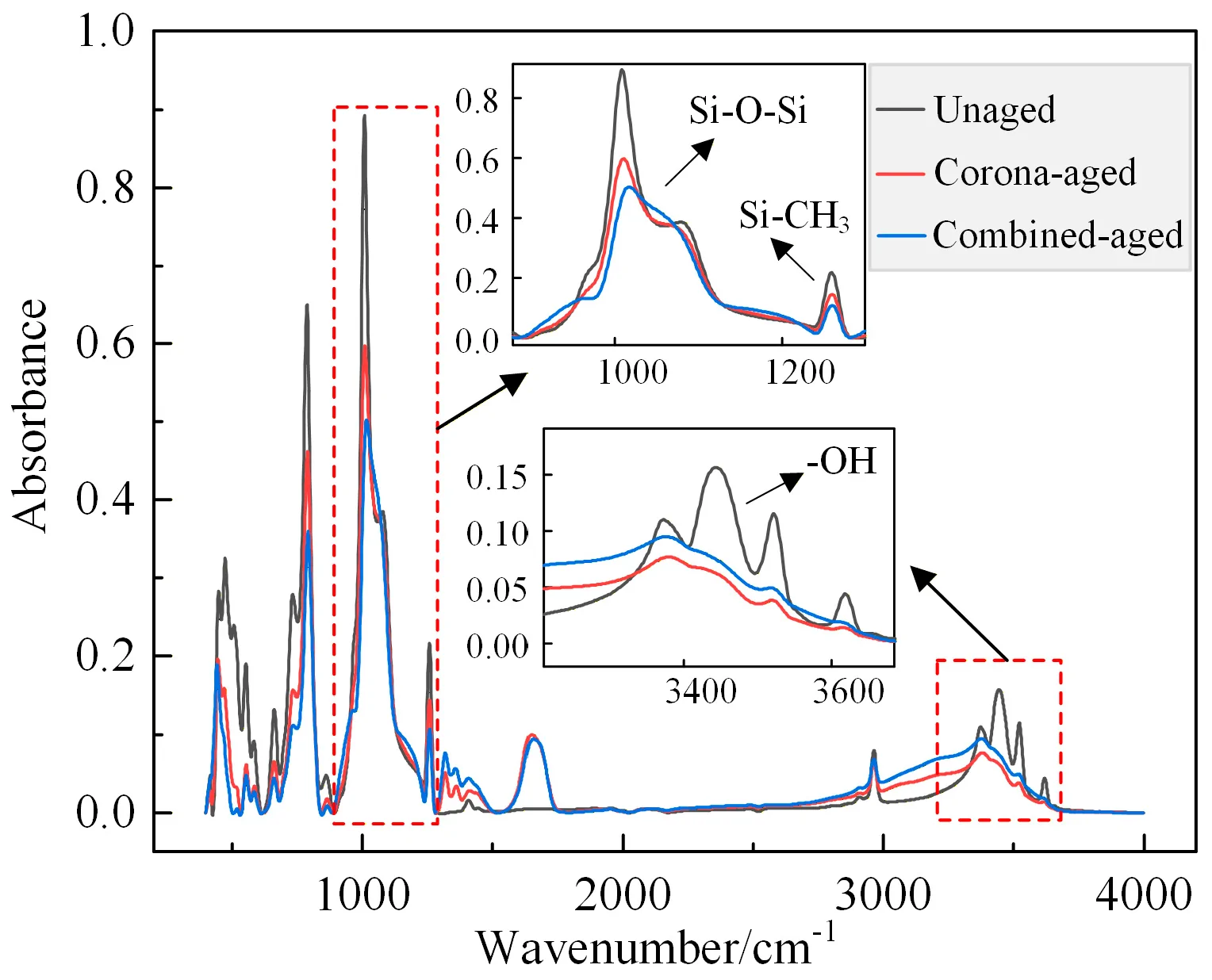
FTIR test results of S3 type specimen.

The chemical structure of the HTV silicone rubber materials was characterized by Fourier transform infrared spectroscopy (FTIR). The test results for the specimens in the initial state, after single corona aging, and after combined erosion–corona aging are shown in Figure 8. After the tests, the FTIR spectra showed a decrease in the absorption peak intensities at 1008 cm^−1^ (Si-O-Si) and at 1260 cm^−1^ (Si-CH_3_), indicating that the high-energy electron beams generated by corona discharge bombarded the material surface and caused scission of both the main chains and side chains of the HTV silicone rubber molecules. An absorption peak of C=O appeared in the 1650–1750 cm^−1^ region, which is a key indicator of the oxidation of silicone rubber molecular chains, indicating that reactive oxygen species generated by corona discharge induced oxidation reactions on the material surface. Before the test, the specimens exhibited a stretching vibration peak of -OH bonds near 3400 cm^−1^, which originated from the filler ATH in the silicone rubber; after aging, the absorption peak intensity of -OH increased. This is because, during the aging process, after the Si-O-Si main chain of the HTV silicone rubber breaks, the Si-O- end groups readily combine with free water to form Si-OH, resulting in an increased absorption peak intensity of -OH. The variation trend of the above functional groups is consistent with the FTIR characteristics of corona-aged silicone rubber reported in existing studies [29].

The peak heights of the absorption peaks were extracted from the FTIR spectra to characterize the intensity of the corresponding functional groups. For the S3 material, after the same corona aging duration, the intensities of the Si-O-Si and Si-CH_3_ absorption peaks decreased by 33.1% and 33.3%, respectively, under single corona aging, whereas they decreased by 43.9% and 50.5% under combined aging. This indicates that the synergistic effect of wind–sand and corona exacerbated the scission of the main chains and side groups of the HTV silicone rubber. Furthermore, because the surface structure of the S3 material remained relatively intact after aging, the reactive oxidizing species generated by corona discharge were effectively trapped within the dense inorganic silica-like layer on the surface, confining oxidation mainly to the shallow surface layer, and a certain amount of C=O groups were still observed on its surface after the test. These chemical changes confirm that wind–sand erosion damage accelerates the corona-induced degradation of silicone rubber molecular chains, thereby expediting the overall aging process.

**Figure 9:**
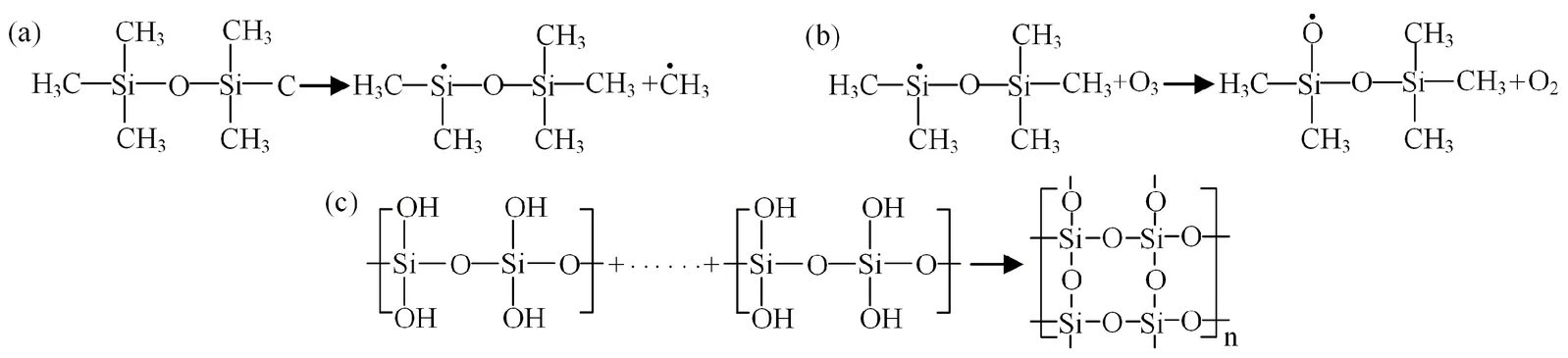
Chemical reactions in the formation of inorganic silica layers: (**a**) side-chain decomposition reaction; (**b**) side-chain oxidation reaction; (**c**) formation of inorganic siloxane layer.

**Figure 10:**
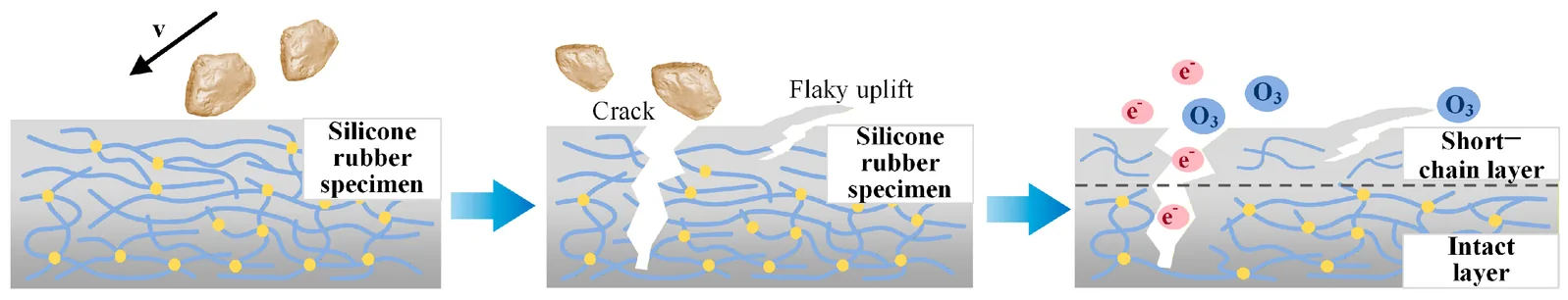
Acceleration mechanism of windblown sand erosion on corona aging.

Based on the above analysis, during corona aging, HTV silicone rubber undergoes molecular chain scission, oxidation, and crosslinking via hydroxyl groups to form an inorganic silicon oxide layer. The relevant chemical reactions are shown in Figure 9. After the molecular chains of HTV silicone rubber undergo scission and degradation, a large number of short-chain molecules and hydroxyl groups are generated. Through these hydroxyl groups, a tighter crosslinking network can form between the molecular chains, in which one silicon atom may be bonded to three or four oxygen atoms, ultimately resulting in an inorganic silicon oxide layer structure on the HTV silicone rubber surface. The formation of this inorganic silica-rich layer leads to a relative increase in the surface Si and O contents and a decrease in the C content after aging, a trend that is consistent with the EDS results. For HTV silicone rubber materials with an intact surface structure, this inorganic silicon oxide layer can serve as a physical barrier, blocking the intrusion of high-energy electrons and reactive species such as ozone, thereby delaying material aging to a certain extent. However, under the effect of windblown sand erosion, the deep and long micro-cracks on the material surface disrupt the integrity of this protective layer, weakening its shielding effect. Consequently, high-energy electrons, ozone, and other species generated during corona discharge can penetrate into the deeper layers of the material through these crack channels, causing aging to propagate rapidly along the erosion paths toward the material interior and greatly accelerating the corona aging process. The above mechanism is illustrated in Figure 10.

**Figure 11:**
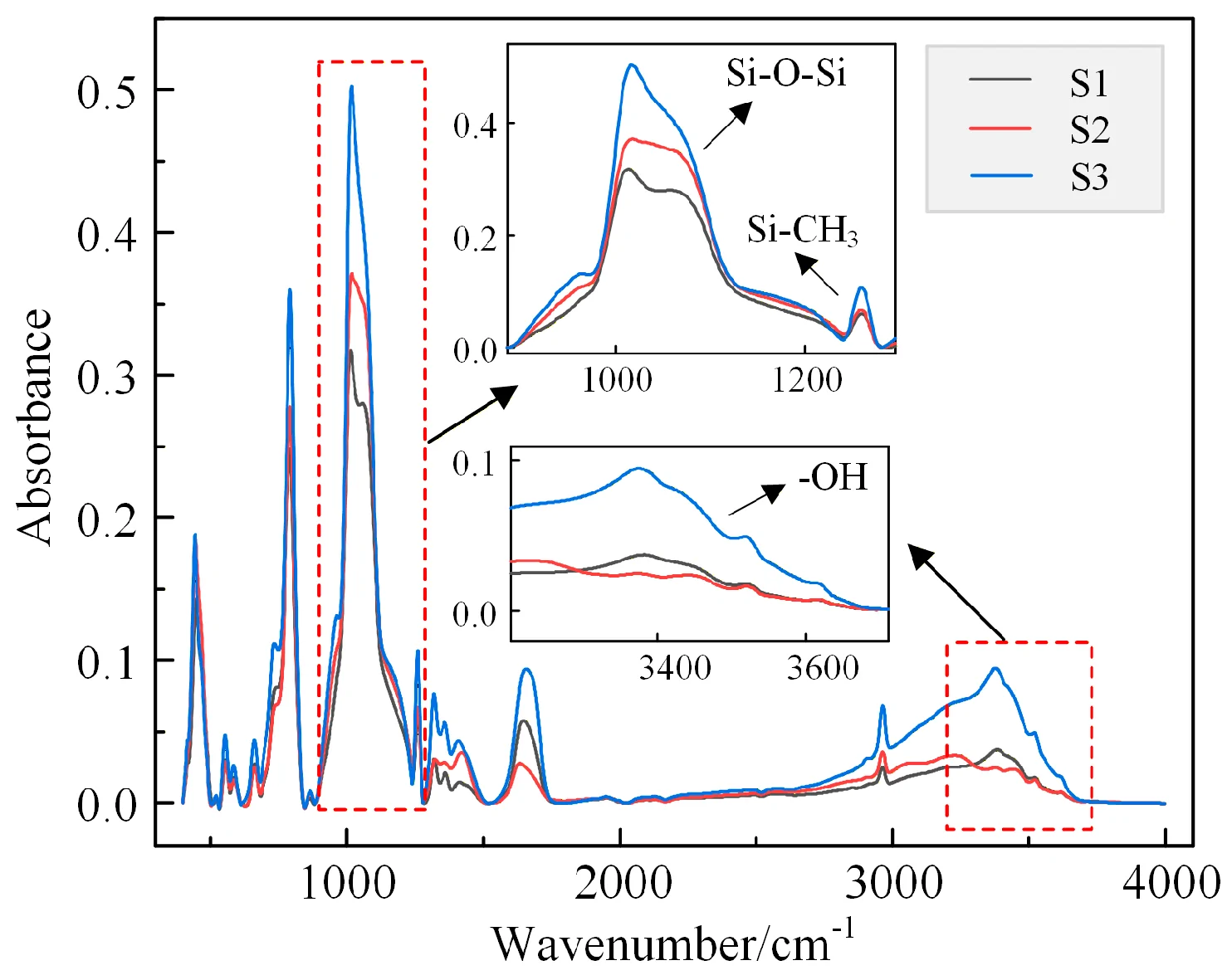
FTIR test results of different silicone rubber materials after combined aging.

To analyze the changes in functional groups of the different HTV silicone rubber materials after the combined aging test, FTIR was performed on the three materials, and the results are shown in Figure 11. After the test, the characteristic peak areas of the Si-O-Si and Si-CH_3_ groups of material S3 were larger than those of the other two materials, indicating that its silicone rubber molecular chains remained relatively intact and that fewer side chain scissions occurred. Because the high-hardness material maintains better structural integrity after erosion, corona aging causes the least damage to its chemical structure, demonstrating superior resistance to both erosion and corona aging.

#### 4.2.3. Hydrophobic Transfer Property

**Figure 12:**
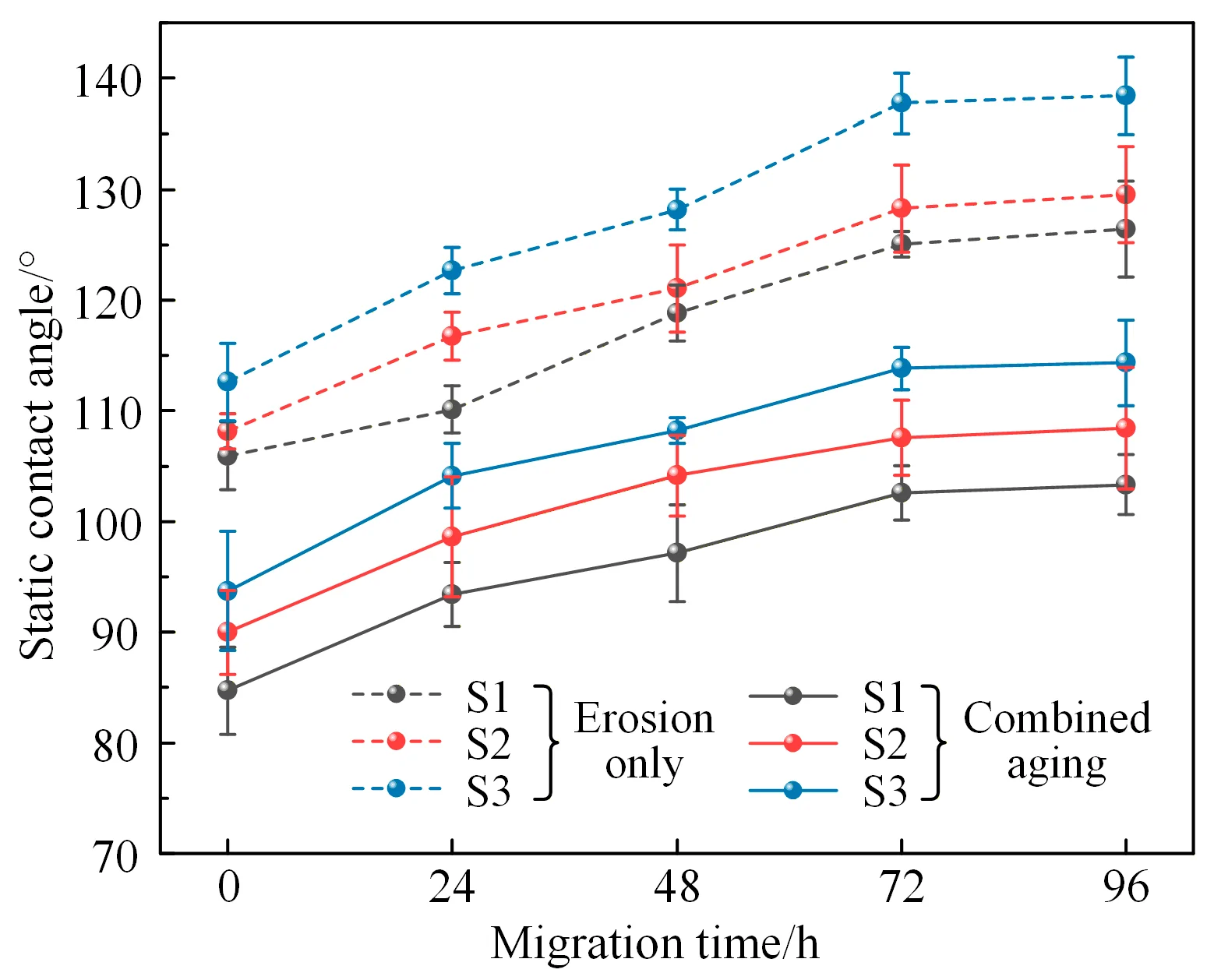
The hydrophobicity transfer curves of different silicone rubber materials.

Figure 12 shows the variation in hydrophobicity transfer behavior of the three silicone rubber materials under single wind–sand erosion and combined aging. After 72 h, the hydrophobicity of the materials tended to stabilize, indicating that the diffusion of low-molecular-weight siloxanes from the interior of the silicone rubber to the external contamination layer was essentially complete, and the low-molecular-weight components had covered the surface of the contamination layer. Under single wind–sand erosion, the static contact angles of S1, S2, and S3 after 72 h of transfer were 125.07°, 128.29°, and 137.75°, respectively. Since wind–sand erosion had relatively little effect on the chemical structure of the silicone rubber, the materials still maintained good hydrophobicity transfer properties. Under combined aging, both the hydrophobicity and the hydrophobicity transfer property of the materials decreased. After 72 h of transfer, the static contact angles of the three silicone rubber materials were only 102.6°, 107.57°, and 113.83°, respectively. This phenomenon can be attributed to the formation of a large amount of cyclic siloxanes with poor migration ability on the material surface under the combined action of erosion and corona aging, leading to a weakening of the hydrophobicity transfer property.

Under the scouring action of the high-speed wind–sand flow, the surface of the S3 silicone rubber material with higher hardness developed pits and flaky protrusions, presenting the densest micron-scale concavo-convex structure overall, which resulted in a higher measured static contact angle than those of the other two materials. The S1 material with lower hardness had a smaller surface roughness and thus exhibited the lowest static contact angle. Under combined aging, the S1 material underwent the most severe aging, leading to a low content of mobile low-molecular-weight siloxanes on its surface; consequently, its steady-state contact angle dropped to the lowest level, and its hydrophobicity transfer ability was severely lost.

#### 4.2.4. Mechanical Properties

**Figure 13:**
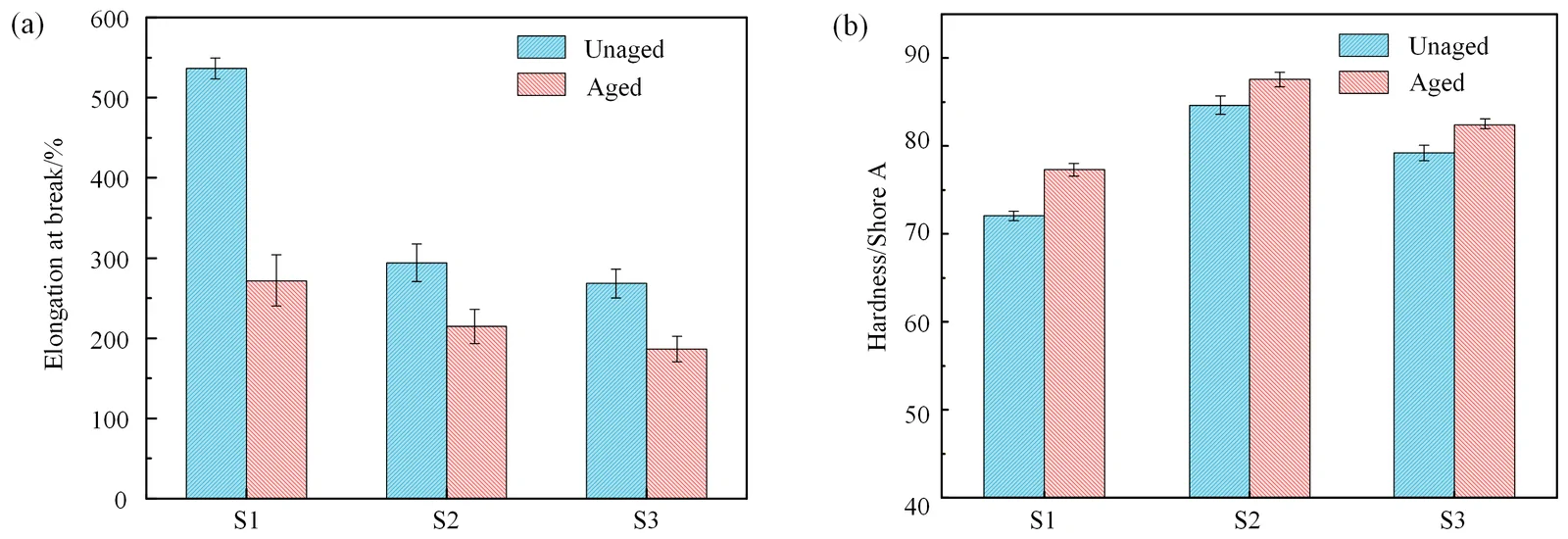
Mechanical properties of different silicone rubbers before and after test: (**a**) Elongation at break; (**b**) Shore A hardness.

Figure 13 shows the elongation at break of the three silicone rubber specimens before and after the tests. After being subjected to sand erosion and then corona aging, the mechanical properties of all specimens decreased. Following the test, the elongation at break of the S1 silicone rubber specimen was 214.83%, representing a decrease of 49.4% compared with its initial state before the test. The elongations at break of the S2 and S3 specimens decreased by 30.4% and 26.9%, respectively, relative to their initial states. The magnitude of decrease in the mechanical properties of the S3 silicone rubber material was only about 50% of that of the S1 material.

The Shore hardness of the HTV silicone rubber specimens was further measured after the tests. The results showed that aging increased the hardness of the HTV silicone rubber materials, with a partial loss of their high-elasticity characteristics. During the corona aging process, the molecular chains of the HTV silicone rubber underwent scission, destroying the crosslinked network structure of the material, leading to surface hardening and a decline in mechanical properties [30]. Meanwhile, the micro-cracks generated during corona treatment caused localized stress concentration in the specimens under tension, further exacerbating the degradation of the mechanical properties. For the lower-hardness S1 silicone rubber material, the dense cracks caused by wind–sand erosion and the micro-cracks induced by corona treatment superimposed on each other, resulting in a significant decline in its mechanical properties.

#### 4.2.5. Surface Resistivity and Dielectric Properties

The surface resistivity of HTV silicone rubber is an important parameter reflecting its insulation performance. In this study, the surface resistivity of the three HTV silicone rubber materials was measured before and after the combined erosion and corona aging, and the results are presented in Table 4. The surface resistivity is reported as the median of the measurements, accompanied by the first quartile, third quartile, and interquartile range. The small interquartile range indicates that the median provides a good representation. After the test, the surface resistivity of the materials decreased significantly. The surface resistivity of the S2 and S1 silicone rubber decreased by four orders of magnitude, while that of the S3 silicone rubber decreased by two orders of magnitude.

The change in the surface chemical structure of the material after the test is the primary cause of the decrease in surface resistivity. The high-energy electron beam generated by corona discharge continuously bombards the HTV silicone rubber surface, causing molecular chain scission and generating a large number of free radicals, which in turn lead to a reduction in surface resistivity. In addition, the cleavage of the HTV silicone rubber main chain produces Si-OH; the -OH group is a polar group that can adsorb moisture from the air, further lowering the surface resistivity. Among the three HTV silicone rubber materials, the aged S3 type exhibited a higher surface resistivity, retaining relatively intact insulation performance.

**Figure 14:**
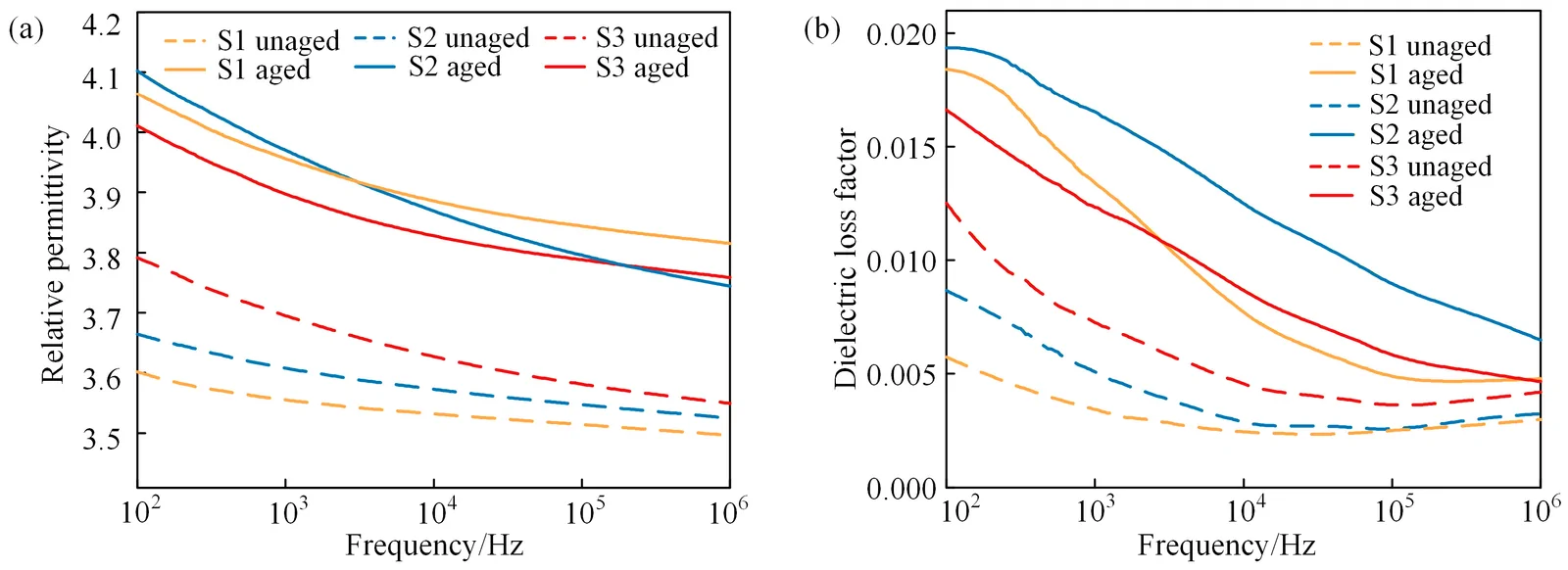
Dielectric properties of silicone rubbers before and after testing: (**a**) relative permittivity; (**b**) dielectric loss factor.

The dielectric properties of the different HTV silicone rubber materials after the same aging duration are shown in Figure 14. In the medium-to-high frequency range, the dielectric constants of the three aged HTV silicone rubber materials remained essentially stable with increasing frequency and were all higher than those of the untreated materials. After the tests, the dielectric loss factor of the materials increased in all cases. In the low-frequency range, taking 100 Hz as an example, the dielectric loss value of the S3 material increased from 0.0125 to 0.0199, while that of the S1 material increased from 0.0057 to 0.0166. In the high-frequency range, taking 10^6^ Hz as an example, the dielectric loss value of the S3 material increased from 0.00419 to 0.00464, while that of the S1 material increased from 0.00298 to 0.00477. The increase in the dielectric loss factor was most pronounced for the lower-hardness S1 material.

The primary polarization mechanisms in HTV silicone rubber are electronic displacement polarization and ionic displacement polarization. The time required for polarization to establish is relatively short and is nearly unaffected by frequency variations; therefore, the dielectric constant of the specimens remains essentially constant over the medium-to-high frequency range. After aging treatment, the scission of the HTV silicone rubber main chain generates a large number of low-molecular-weight products and free radicals. These small-molecule free radicals are highly polarizable under an electric field, leading to an increase in the relative permittivity of the material. Accompanying the generation of free radicals during aging, the concentration and mobility of charge carriers in the material increase, resulting in elevated conduction loss and an increase in the dielectric loss facto [31]. According to the FTIR results for the three HTV silicone rubber materials before and after testing, the S1 material exhibited the highest degree of oxidation and chain scission reactions, causing its dielectric constant and dielectric loss to increase substantially relative to the unaged state.

### 4.3. Selection Method of HTV Silicone Rubber for Composite Insulators in Desert Regions

Table 5 presents the degree of degradation in the mechanical and electrical properties of the three materials after windblown sand erosion and combined erosion–corona aging, expressed as the percentage decrease in each property relative to the initial state. Under the effect of wind–sand erosion alone, the S1 material with an initial hardness of 72.1 Shore A exhibited obvious structural damage on its surface, and its micro-mechanical properties such as nano-hardness and Young’s modulus, as well as macro-mechanical properties such as elongation at break and tensile strength, all decreased significantly. In contrast, the S2 material with an initial hardness of 79.2 Shore A and the S3 material with an initial hardness of 84.6 Shore A maintained relatively good structural integrity after erosion, and the decreases in both micro- and macro-mechanical properties were smaller than those of the S1 material, exhibiting stronger resistance to wind–sand erosion. The intact surface structure after erosion not only preserved the mechanical properties of the material but also provided a physical barrier against corona aging in the subsequent service environment.

For the S1 material, whose surface had already developed dense cracks after windblown sand erosion, the damaged areas would serve as ingress pathways for reactive species such as high-energy electrons and ozone during the corona aging process, causing aging to propagate rapidly along the erosion fissures toward the interior of the material and significantly accelerating the overall aging process. A comparison of the properties of the three materials after aging indicates that, although the S1 material possesses superior initial mechanical properties, its performance degradation rate under combined aging conditions is much higher than that of the other two materials, making it unsuitable for long-term service in desert regions. Comparing the other two materials, the S3 material, owing to its ability to maintain relatively high structural integrity after erosion, exhibited the smallest magnitude of degradation in all properties after the same duration of erosion and aging. In particular, the degradation in its electrical properties after aging was less than 50% of that of the S1 material, demonstrating its excellent adaptability to sandstorm-prone environment. Considering the synergistic effect of sandstorm and corona discharge, the S3 higher-hardness materials are more suitable for the sandstorm-prone environment of desert regions. Therefore, when selecting HTV silicone rubber for composite insulators in such regions, it is recommended that materials with higher hardness be preferentially chosen, provided that the basic mechanical performance requirements are met, in order to enhance their resistance to multi-factor synergistic aging in sandstorm-prone environment.

## 5. Conclusions

This paper investigates the surface degradation mechanisms of three HTV silicone rubber materials under single windblown sand erosion and under the synergistic effect of erosion and corona aging, and compares the degradation characteristics of their electrical and mechanical properties. The main conclusions are as follows:

(1) Under single windblown sand erosion, the formation of surface micro-cracks and spalling layers is the primary cause of the decline in multi-scale mechanical properties such as nano-hardness and elongation at break. The lower the hardness of the HTV silicone rubber, the more readily its surface structural integrity is compromised during sand particle impact, and the weaker its erosion wear resistance.
(2) Windblown erosion exerts an accelerating effect on corona aging. Under the synergistic effect of erosion and corona aging, high-energy electrons and ozone molecules generated by corona discharge penetrate deep into the material along the fissures created by erosion, causing aging to propagate in depth and accelerating the corona aging process.
(3) The hardness of HTV silicone rubber materials is positively correlated with their corona aging resistance after erosion. Among the three tested materials, the S3 silicone rubber retained relatively intact micro-morphology and molecular structure after combined aging treatment, exhibited the smallest degradation in mechanical and electrical properties, and demonstrated the best environmental adaptability to desert regions. It is recommended that the S3 silicone rubber be preferentially selected as the sheath material for composite insulators in such regions.

Under the experimental conditions employed in this study, where sand erosion was applied first and corona aging subsequently, the silicone rubber material with higher hardness exhibited superior resistance to synergistic aging. Therefore, for the selection of composite insulators intended for desert regions, it is recommended that, on the premise that the basic mechanical properties meet the required standards, silicone rubber materials with higher hardness be given priority, such as those with initial properties similar to S3, so as to delay the synergistic aging effect of sand erosion and corona discharge and to enhance the operational reliability of insulators in such environments. Given the limitations of accelerated aging tests in terms of reproducing environmental factors and test duration, future work can further explore the applicability of this recommendation under different aging sequences and actual complex service conditions, and verify the long-term service performance of the S3 material through field trials on operating power lines, so as to provide a more comprehensive scientific basis for extending the service life of composite insulators in desert regions.

## Figures and Tables

**Table 1:** Formulations of the three silicone rubber materials.

Type	Methyl Vinyl Silicone Gum/Phr	Alumina Trihydrate/Phr	Fumed Silica/Phr
S1	100	122~128	28~32
S2	100	117~123	33~37
S3	100	112~118	36~40

**Table 2:** Initial performance parameters of different HTV silicone rubber materials.

Type	Hardness/Shore A	Tensile Strength/MPa	Elongation at Break/%	Tear Strength/(N∙mm^−1^)
S1	72.1	5.35	536	16.55
S2	79.2	5.53	287	11.20
S3	84.6	5.40	308	13.90

**Table 3:** The atomic percentage of main elements on the surface of the sample.

Element Content/%	Pre-Combined Aging			Post-Combined Aging		
	S1	S2	S3	S1	S2	S3
C	42.75	43.26	44.77	12.79	10.64	15.32
O	25.29	26.34	27.12	39.53	44.07	41.33
Si	21.03	21.86	21.38	37.52	37.26	36.84
Al	10.93	8.54	6.73	10.16	8.03	6.51

**Table 4:** Surface resistivity of sample.

Type	Resistivity Before Aging (Ω·m)	Q1	Q3	IQR	Resistivity After Combined Aging (Ω·m)	Q1	Q3	IQR
S1	3.41 × 10^15^	3.32 × 10^15^	3.49 × 10^15^	1.70 × 10^14^	9.51 × 10^11^	9.40 × 10^11^	9.65 × 10^11^	2.50 × 10^10^
S2	2.89 × 10^15^	2.75 × 10^15^	2.93 × 10^15^	1.80 × 10^14^	8.21 × 10^11^	8.11 × 10^11^	8.34 × 10^11^	2.30 × 10^10^
S3	2.97 × 10^15^	2.84 × 10^15^	3.06 × 10^15^	2.20 × 10^14^	9.82 × 10^13^	9.69 × 10^13^	9.98 × 10^13^	2.90 × 10^12^

Surface resistivity is expressed as the median of the test results; Q1 is the first quartile, Q3 is the third quartile, and IQR is the interquartile range.

**Table 5:** Property changes in different materials after sand erosion and combined sand–corona aging.

Type	Wind–Sand Erosion				Combined Sand–Corona Aging			
	Nano-Hardness	Young’s Modulus	Elongation at Break	Tensile Strength	Elongation at Break	Surface Resistivity	Relative Permittivity	Dielectric Loss Factor
S1	24.10% (↓)	23.71% (↓)	28.48% (↓)	15.50% (↓)	49.39% (↓)	4 orders of magnitude (↓)	12.83% (↑)	191.23% (↑)
S2	21.02% (↓)	14.24% (↓)	15.91% (↓)	9.21% (↓)	30.45% (↓)	4 orders of magnitude (↓)	11.96% (↑)	113.95% (↑)
S3	19.38% (↓)	11.61% (↓)	17.99% (↓)	10.88% (↓)	26.88% (↓)	2 orders of magnitude (↓)	5.79% (↑)	59.20% (↑)

(↑) indicates an increase in the property value, and (↓) indicates a decrease.

## Data Availability

The original contributions presented in this study are included in the article. Further inquiries can be directed to the corresponding author.
